# When You Watch Your Team Fall Apart – Coaches’ and Sport Psychologists’ Perceptions on Causes of Collective Sport Team Collapse

**DOI:** 10.3389/fpsyg.2019.01331

**Published:** 2019-06-12

**Authors:** V. Vanessa Wergin, Clifford J. Mallett, Christopher Mesagno, Zsuzsanna Zimanyi, Jürgen Beckmann

**Affiliations:** ^1^ Department of Sport and Health Sciences, Chair of Sport Psychology, Technical University of Munich, Munich, Germany; ^2^ Faculty of Health and Behavioural Sciences, School of Movement and Nutrition Sciences, University of Queensland, Brisbane, QLD, Australia; ^3^ School of Health and Life Sciences, Federation University Australia, Ballarat, QLD, Australia

**Keywords:** collective team collapse, emotional contagion, key player collapse, performance contagion, team choking

## Abstract

Collective team collapse occurs when multiple players of a sport team experience a sudden and extreme underperformance within a game and are unable to return to their initial performance level. The occurrence of such a team collapse event commonly leads to the loss of the game or championship. A recent study investigated athletes’ perceptions of the phenomenon and proposed a process model of causes of collective sport team collapse. The main goal of this study was to apply this process model to the data collected from coaches and sport psychologists. A further goal was to explore differences in perceptions of causes of team collapse among athletes, coaches, and sport psychologists of various professional German sport teams. Semi-structured interviews were conducted to investigate seven coaches’ and four sport psychologists’ perceptions. Following an abductive approach, a deductive content analysis was used to explore if the data supported the process model of collective sport team collapse. Perceived antecedents and critical events causing team collapse were similar among the three participant groups. Coaches and sport psychologists differed from athletes in their perception of emotional, cognitive, and behavioral outcomes of team collapse. Coaches tended to report behavioral factors, such as immobility or the blaming of other players, as critical factors maintaining team collapse. Sport psychologists reported cognitive factors, such as individualization or a lack of accountability between the players, to be relevant for team collapse maintenance. Overall, the data of this study supported the general structure of the process model of collective sport team collapse; however, minor amendments to the temporal cascade of causes of team collapse are introduced. Future research is encouraged to examine this model, to provide guidance to teams, coaches, and sport psychologists in dealing with collective sport team collapse.

## Introduction

“I’m past it, but I’m not over it. I don’t think I’ll ever be.” (Orr, 2017) was what head coach Dan Quinn said a few weeks after the Atlanta Falcons dramatically lost the 2017 Super Bowl. Their sudden underperformance is often referred to as a collective team collapse, since they led 28-3 during the second half of the game but eventually lost 28-34. Collective team collapse can be defined as “a sudden, collective, and extreme underperformance of a team within a competition, which is triggered by a critical situation that interferes with the team’s interplay, a loss of control of the game, and ultimately the inability of the team to regain their previous performance level within the game.” (Wergin et al., 2018, p. 15).

Although collective sport team collapse is a widely known phenomenon, research investigating its causes is lacking. In an initial case study, Apitzsch (2009a) investigated causes of collective team collapse in nine male handball players of the same team and found that inappropriate behavior, failure of the role system, negative communication, a change in the opponents’ tactics and goals scored by the opponent were factors that played a role in the specific collapse that was described by players of the handball team. He further reported that negative thinking, negative emotions, and negative emotional contagion should be dealt with in order to prevent a team collapse. In a further study with athletes and coaches, Apitzsch (2009b) explored, in part, four male (handball, ice hockey, and soccer) coaches’ perceptions on collective team collapse. Similar to the first study, Apitzsch reported coaches’ perceptions of the major causes of team collapse to be inappropriate behavior, a failure of a team’s role system, negative communication, a change in the tactics of the opponent, and the opposition scoring points. Moesch and Apitzsch (2012) interviewed nine female elite handball coaches about their perceptions of positive and negative psychological momentum. Psychological momentum is defined as “a change in cognition, affect, physiology, and behavior caused by an event or series of events that will result in a commensurate shift in performance and competitive outcome” and can be either positive or negative (Taylor and Demick, 1994, p. 54). Moesch and Apitzsch (2012) found that negative psychological momentum was associated with various factors related to coach and individual players, such as passive coaching behavior or anxiety and stress in the players. Furthermore, Moesch and Apitzsch reported confidence as well as external factors (e.g., referee decisions) or team factors, such as not taking responsibility for what happens on the court, to be related to negative psychological momentum.

Although there are good initial studies on athletes’ and coaches’ perceptions of team collapse and psychological momentum, some limitations exist. For example, Apitzsch’s (2009a) study investigated causes of team collapse in a case study design. In order to explore the causes of the phenomenon of team collapse and draw general conclusions, different game situations and various types of sport are needed. Furthermore, Apitzsch’s (2009a,b) qualitative studies were conducted without audiotaping and transcription of the interviews; instead notes were taken by the researchers, which may have limited their abilities to fully engage in the interview and thoroughly follow the statements made. The interview guides and methods of analysis used in the studies are rarely described, which limits transparency and complicates the conduction of subsequent studies. Besides that, Apitzsch’s (2009a,b) studies involved only male athletes and coaches and Moesch and Apitzsch’s (2012) study included exclusively female handball coaches, which limits researchers’ ability to draw conclusions among gender. Moreover, Moesch and Apitzsch explored causes of psychological momentum rather than collective sport team collapse. A further improvement suggestion is that perceptions of other coaching staff, who are less involved in the game, and therefore have a more distanced view, than the coach, should also be included since they provide other perspectives about team collapses in sport.

**Figure 1:**
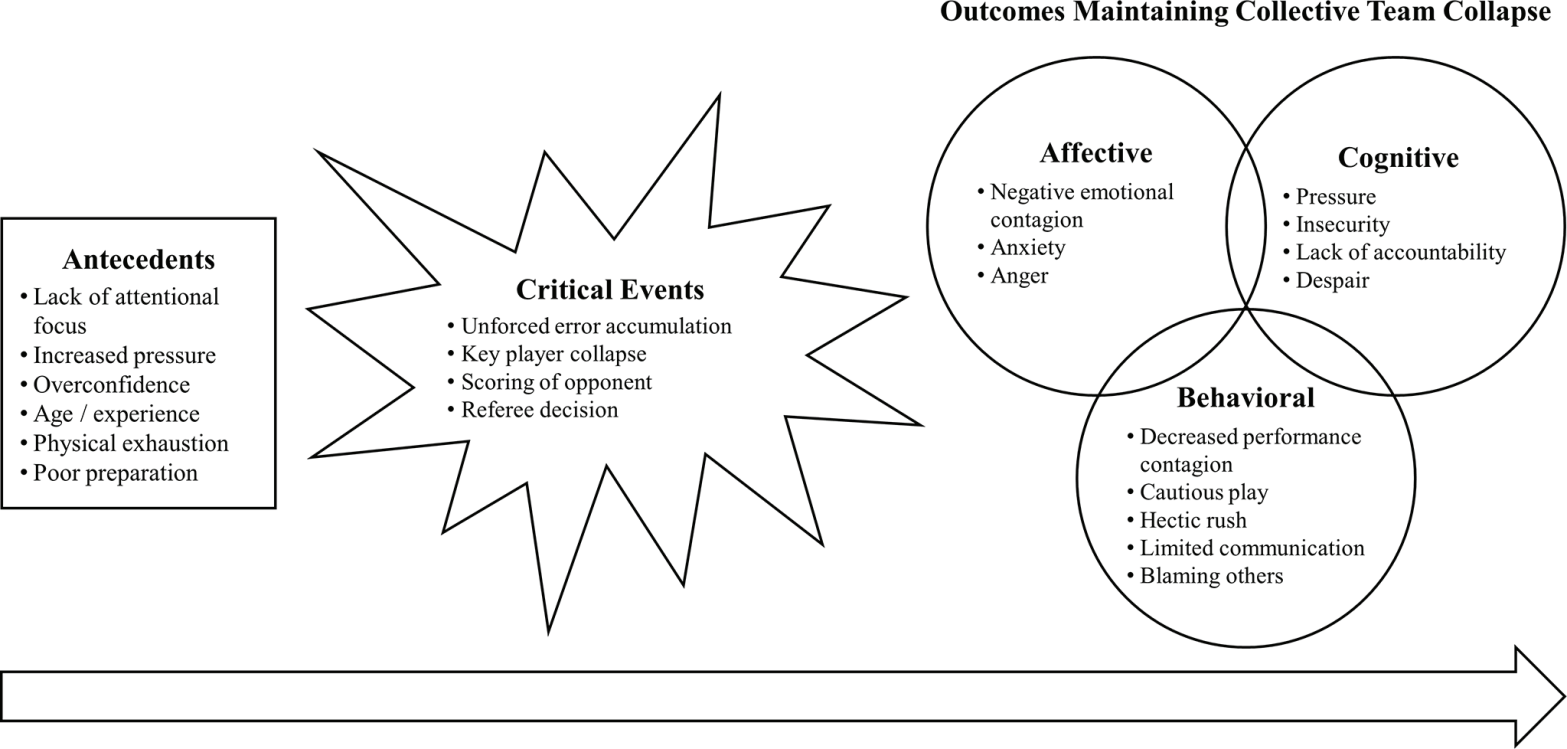
Process model of causes of collective sport team collapse (Wergin et al., 2018).

Another issue regarding existing research in the field is that many studies tend to employ interchangeable terms to describe the collective collapse of a sport team. Especially, negative momentum is a term widely used in sport psychology research (e.g., Crust and Nesti, 2006; Den Hartigh et al., 2014; Moritmer and Burt, 2014) to describe shifts in a team’s performance. In order to distinguish between the terms of negative momentum and collective team collapse and to gain first insights into causes of collective team collapse of different teams in various sport, Wergin et al. (2018) conducted a qualitative study. Within this study, 10 team sport athletes from various teams and sports were interviewed on their perception of causes of a collective team collapse they had experienced with their team. Results indicated that collective team collapse was induced by a temporal cascade of causes rather than by single triggers. This cascade included antecedents (i.e., factors that make the occurrence of a team collapse more likely), critical events (i.e., specific occasions within the game) as well as affective, cognitive, and behavioral outcomes that foster a maintenance of the team collapse. Within Wergin and colleagues’ theoretical framework, social factors, such as decreased performance contagion or emotional contagion, played crucial roles in causing and maintaining team collapse, illustrating that team collapse seems to be more than concurrent choking of several individual players. These findings were compiled in a process model of collective sport team collapse (Figure 1). Based on these results, Wergin et al. (2018) distinguished collective team collapse from the term negative momentum: Collective team collapse is described to be chronic and more extreme than negative momentum. It is accompanied by an inability of the team to return to previous levels of performance. In contrast to negative momentum, a collective team collapse does not shift between teams, but causes one team to underperform dramatically during competition. While negative momentum can be related to both individual and team-based sports, collective team collapse is a term specifically describing sudden and dramatic underperformance of sport teams.

Wergin et al.’s (2018) finding provides first insights into causes of collective collapse and some clarity for the differentiation between the terms collective team collapse and negative momentum. One restriction of Wergin et al.’s (2018) study, however, is that only athletes’ perspectives were included. In order to gain a more global view of the phenomenon, other observers’ (e.g., coaches, sport psychologists, or officials) perceptions of the team collapse event should also be considered. Thus, the main goal of the current study was to explore coaches’ and sport psychologists’ perceptions of causes of collective team collapse across different sports. Coaches and sport psychologists would offer two different observational perspectives of the phenomenon of collective sport team collapse and would enhance our understanding of the phenomenon. A second goal was to qualitatively compare coaches’ and sport psychologists’ perspectives to athletes’ perceptions reported by Wergin et al. (2018). The third goal was to explore whether coaches’ and sport psychologists’ perceptions would support the process model of causes of collective sport team collapse, that was developed based on athletes’ perceptions of team collapse.

## Materials and Methods

### Philosophical and Methodological Orientation

A relativist ontology and a constructivist epistemology (Ritchie et al., 2013; Sparkes and Smith, 2014) were considered most appropriate to investigate coaches’ and sport psychologists’ interpretation of causes of collective team collapse. A relativist ontology assumes that humans develop subjective mental constructions of reality (Sparkes and Smith, 2014). These constructions of reality can be understood and interpreted using a constructivist epistemology, which considers that the interpretation of data relies not only on the participant’s subjective interpretation of reality, but also the researcher’s interpretation of the participant’s perspective, which is influenced by the interaction between the participant and the researcher’s ontological approach.

Since the process model of collective team collapse was applied as a theoretical framework to the data collected in this study, abductive reasoning was used as a method of data analysis (Peirce, 1960/1979). Abduction is understood as a form of pragmatism, which favors practical action over theoretical reason (Timmermans and Tavory, 2012). Abduction is used to examine the fit between existing hypotheses or theories and current data. As a result of abduction, existing theories can be modified, rejected, or elaborated upon to explain the data (Kennedy and Thornburg, 2018). The approach constantly compares theory and data and requires an openness to both data and preexisting theories in order to incorporate the two. Abduction assembles the advantages of inductive and deductive approaches, as, in contrast to inductive approaches, it is guided by a theory and prohibits “wild guessing,” and, in contrast to deductive approaches, it is open to the change of existing theories for the sake of representing the data as well as possible.

### Participants

The sample (*N* = 11) consisted of seven coaches (five male) and four sport psychologists (all male) of different team sports. Two coaches and one sport psychologist had a background in volleyball, two coaches and one sport psychologist in soccer, two coaches in basketball, two sport psychologists in handball, and one coach in field hockey. Participants’ age ranged from 25 to 55 for coaches (*M* = 34.14, *SD* = 10.80) and from 38 to 55 for sport psychologists (*M* = 45.50, *SD* = 8.35). All participants were coaching either the German national team of their sport or a team playing in between first and fourth divisions in Germany. This competitive level of teams, coaches, and sport psychologists was required to ensure that the teams’ collapses were not a result of a lack of skills in athletes, coaches, or psychologists. Inclusion criterion for selection was that participants had to have worked as a coach or sport psychologist for at least 10 years. Participants’ actual experience ranged between 11 and 44 years for coaches (*M* = 24.57, *SD* = 9.93) and between 10 and 28 years for sport psychologists (*M* = 17.00, *SD* = 8.37). The time they were working with their current team varied between 0.5 and 5 years for coaches (*M* = 2.00, *SD* = 1.61) and between 0.3 and 12 years for sport psychologists (*M* = 5.21, *SD* = 4.93).

### Interview Guide

The interview questions were based on Wergin et al.’s (2018) interview guide. Accordingly, participants were initially asked to report a team collapse they had experienced with their team based on a short colloquial description of the phenomenon of team collapse: “A collective team collapse is the moment or process, when the performance of your team unexpectedly decreases more than normal. It is the situation, when your team experiences a significant performance collapse during a competition/game. It is the moment or process when ‘nothing works anymore’ within your team during a specific competition/game.” Afterward, they answered a section of questions about details of the team collapse (questions 1–7); for example, “How many players were involved?” or “At what point during the game did the collapse occur?” The next section contained questions about the impact the collapse had on the team (questions 8–12); for example, “To what extent did the team collapse influence the further course of play?” The final question (question 13) dealt with specific triggers of team collapse: “In your opinion, what were the influencing factors for the team collapse?” Participants were then asked whether there was anything else they would like to add related to team collapse (question 14). The full interview guide is included in Supplementary Data Sheet 1.

### Data Collection

The study did not involve any invasive or potentially dangerous methods and therefore, in accordance with the German Research Foundation (DFG) and the guidelines of the Department of Sport and Health Science at the Technical University of Munich, did not require formal ethical approval. Participants were recruited through the purposive sampling method of criterion-based sampling. Coaches and sport psychologists were recruited when they fulfilled the following criteria (similar to Wergin et al., 2018): (1) being a coach or sport psychologist of a team sport consisting of more than two players, (2) coaching a team between the first and fourth division, (3) having experience in coaching/applied sport psychology of 10 years or more, (4) having experienced a team collapse event with their current team, and (5) being willing to talk about the team collapse event. They were contacted *via* email or telephone and asked, whether they fulfilled the criteria mentioned above. If they did, they were invited to participate in the study. Recruited participants were informed of voluntary participation, the purpose of the study, and the confidential treatment of data prior to the start of the interview. They were assured the right to quit the interview at any time without penalty. Participants were further informed that audio records would be used for research purposes only and that recorded data would be treated confidentially. Additionally, they signed a declaration of consent, stating that they had been informed about the purpose of the study and agreed with audiotaping of the interview. All participants gave written informed consent in accordance with the Declaration of Helsinki. The retrospective semi-structured interviews were conducted at the sports facilities where participants worked or in one case in course of a seminar. The duration of interviews ranged from 32 to 57 min (*M* = 37.21; *SD* = 7.22).

### Data Analysis and Trustworthiness

The 11 interviews were audio taped and transcribed verbatim by the first and fourth authors, who typed out the recordings manually, yielding 122 total pages of single-spaced text; coaches’ interviews generated 81 pages and sport psychologists’ interviews generated 41 pages. The first and fourth authors read all interview transcripts several times to familiarize themselves with the content. A deductive content analysis was conducted to apply the process model of collective sport team collapse (Wergin et al., 2018) to the experiences reported by coaches and sport psychologists. For example, coaches’ and sport psychologists’ statements, such as “If we would have won this game, we would have been first in the ranking,” were linked to categories of the process model, in this case *increased pressure*. Simultaneously, an inductive content analysis was employed to screen transcripts for novel content. If statements were not represented through preexisting categories of the process model, new categories were developed in accordance with the data, such as *immobility* as a category for the statement “Nothing works anymore, no reception, no movement towards the ball, no extra movement, no reaction, they’re just looking at each other.”

Data collection and analysis were conducted following the recommendations of Smith and McGannon (2018) for developing rigor in qualitative research. Accordingly, member checking, inter-rater reliability, and the notion of universal criteria, which constitute former methods for the development of methodological rigor, were renounced in the analytical process since they were “shown to be ineffective for verification, trustworthiness, or reliability purposes” (Smith and McGannon, 2018, p. 1). Instead, the formal methodological steps of revisiting, defamiliarization, and alternative casing for enriching deductive analysis proposed by Timmermans and Tavory (2012) were followed. In order to “revisit” the phenomenon of team collapse, transcripts, codes, and memos developed during the coding process were reevaluated and rethought several times during the process of data analysis. Furthermore, it can be assumed that the researcher’s ability to see data from different angles and to pay attention to details that would vanish in a regular conversation is enhanced by the textual mode of transcripts. The text and the inscriptions create a “semantic distance from the taken for granted” (Timmermans and Tavory, 2012, p. 177). The methodological step of alternative casing requires the researcher to find as many ways as possible to understand the data. In order to fulfill this methodological step, constant comparisons were used throughout analytical process to compare new data with the theoretical framework of the process model of team collapse. It was explored whether new data conformed to the model and whether the model could explain variation in the data. Once data analysis was finished, the third author, who was not involved in the initial analysis process, acted as a “critical friend” (Sparkes and Smith, 2014; Smith and McGannon, 2018), who challenged the categories as well as the adapted model, and provided independent feedback from an external expert perspective. The final categories and adaptions to the model were discussed extensively among all authors until consensus was reached.

## Results and Discussion

We analyzed coaches’ and sport psychologists’ perception of causes of collective team collapse by applying Wergin et al.’s (2018) theoretical framework, provided through the process model of collective sport team collapse, to the data. Thus, results are presented separately for the three temporal sequences of antecedents, critical events, and outcomes maintaining collective team collapse, as proposed by Wergin et al.’s process model. Within this theoretical framework, categories are presented in the same order as in Wergin et al.’s process model. Results are then compared to athletes’ perceptions in the Wergin et al. study as well as to other collective team collapse research. General differences between athletes’, coaches’, and sport psychologists’ perceptions are discussed in the “General Discussion” section.

### Antecedents

The first antecedent of collective team collapse found by Wergin et al. (2018) is a *lack of attentional focus*. Coaches in the current study also reported this antecedent to be present prior to a team collapse game. Coach 6 (basketball), for example, explained a team collapse in the second half of the game by saying: “In my opinion, it was more or less the whole [third] quarter, it is hard to define… Maybe it was not as bad at the very beginning and [the team] started collapsing after the first minute but often there is this phase: After half time you are in a low, not really awake and the others are motivated and induce pressure and you are in deep sleep and that’s why it doesn’t really work.” Similar to what athletes in the Wergin et al. (2018) study reported, the whole team seemed to be unfocused prior to the team collapse already, which appears to have increased the chances of a motivated opponent scoring and the likelihood of the own team to cause errors. A lack of concentration is also associated with individual choking (Eysenck et al., 2007; Oudejans et al., 2011; Fryer et al., 2017). Complementary to this finding, Morgan et al. (2013) found resetting a team’s focus to increase the team performance and reduce choking under pressure. Resetting a team’s focus may function as a protective factor against choking under pressure or collective team collapse.

Coaches and sport psychologists also found that the perception of *increased pressure* prior to a game made their team more vulnerable to a team collapse. Coach 3 (volleyball), for example, explained the pressure before the game was likely due to ranking if they won: “If we would have won this game, we would have been first in the ranking.” Sport Psychologist 4 (handball) similarly described that his team was under pressure due to the importance of the game: “It was the critical game determining whether we could see the championships as a success or a failure. The team said before [the game] that the quarter finals were their goal and that would have meant a qualification for a major tournament. And in this round of 16 the team had to play against a team, which is one of the best teams in the world in women’s handball.” It appears that perceived importance of the game caused the experience of pressure in team members, which is similar to athletes’ perceptions of antecedents of team collapse (Wergin et al., 2018). These findings support Marchant et al.’s (1998) model of competitive anxiety, which indicates that perceived importance of a game may lead to increased pressure and anxiety in athletes. We assume that perceived pressure caused increased anxiety in players and thereby an increased vulnerability to team collapse. This association between anxiety and failure can also be found in choking under pressure literature (e.g., Hill et al., 2009; Otten, 2009; Mesagno et al., 2012).

Furthermore, coaches and sport psychologists described that their teams’ as well as their own *overconfidence* about winning the game was another common factor preceding a team collapse. Sport Psychologist 1 (soccer) explained that his team lost focus in the game because they thought they had won the game already: “A main indicator [of the team collapse] was that, to exaggerate a little, a few players on the team were busy thinking about the next game already, because they seemed to have ticked off [the win for] that game already, since everything was in such a flow. And the main indicator then was that at least two players did by far not show the same running performance anymore and did not close the spaces anymore.” Coach 5 (basketball) described how he was overconfident about winning the game and accordingly changed the formation of the team to give younger and inexperienced players the chance to play, who then underperformed: “We were in the lead by 20 points five minutes before the end of the game and then you usually assume that you have won the game. I let the other two [younger and inexperienced players] play. Well, first, I substituted one player, then the second. I’m not sure if that was the trigger but it was definitely a disadvantage and you learned that a basketball game is still open two minutes before the end of the game, even if you’re in the lead by 10 or 15 points.” Overconfidence of players or the team was reported as an antecedent in existing research (e.g., Apitzsch, 2009a; Hill and Shaw, 2013; Wergin et al., 2018). Wergin et al. (2018) suggested that overconfidence might cause an overestimation of players’ own abilities and reckless behavior in athletes, leading to failure as a consequence. Overconfidence of a coach has not been reported as an antecedent of team collapse in existing literature so far. It is not our intent to jump to any conclusions based on this single case, but the possibility cannot be ruled out that, similar to athletes’ overconfidence, the coach’s overconfidence may also cause a slightly negligent coaching behavior, characterized by the substitution of successful players with less experienced and younger players, who were not able to deliver their regular performance.

Coaches also reported the antecedent of composition of *age and experience* to be relevant for the occurrence of team collapse. Specifically, young and less experienced players were described to be more vulnerable to the experience of team collapse. Coach 5 (basketball) explained these players tend to have difficulties reacting to tactical changes of the opponent: “If the opponent somehow changes strategy and the team is very young and inexperienced and moves into a negative hole, insecurity develops through this inexperience. That’s how these negative runs happen, it’s related to age and the situation.” Researchers have similarly reported a lack of experience as an antecedent for a bad performance (Moesch and Apitzsch, 2012) and for team collapse (i.e., Apitzsch, 2009a; Wergin et al., 2018). Further research showed that repeated experience of challenging or stressful situations fosters resilience to stress over time (Fletcher and Sakar, 2012; Morgan et al., 2015; Decroos et al., 2017) and assumed that less experienced athletes are more likely to choke in stressful situations.

Coaches further described the antecedent of *poor preparation*. Coach 6 (basketball) explained that his team did not warm up enough during halftime, which made them vulnerable to a team collapse: “After the first half, we talked quickly about what was good and bad and then they went to their friends and chatted with them. They only warmed up for two, three minutes before the end of half time and in my opinion that was a little too late and too short. And they switched off their heads completely during halftime and were in a totally different focus instead of gathering together somewhere and starting to warm up earlier for the second half.” From this description, it appears that spending time with friends distracted the team from the game and changed their focus at the beginning of the second half. This is similar to the findings of Hill and Shaw (2013) and Wergin et al. (2018), but adds to existing research by making a connection between insufficient physical and mental preparation for the game and a lack of attentional focus at the beginning of the game as antecedents of collective team collapse. Similarly, a thorough preparation was found to be of advantage when facing difficult game situations (Morgan et al., 2013; Decroos et al., 2017). This adds to the process model of collective team collapse and will be further discussed in the “General Discussion” section.

Furthermore, *respect for the opponent* was reported as an antecedent in sport psychologists’ transcripts. Sport Psychologist 3 (handball) explained that in his perception, too much respect for the opponent may have led to a lack of self-confidence in their own team: “I was told that, before the game, the team said: ‘In previous games, we were more focused on ourselves and today we talked more about the opponent.’ In the end they thought that was a failure, to talk about the opponent that much and that this was the reason why the awareness of the own strength wasn’t there. And I believe this founded the basis that made the team so vulnerable for this triggering moment [when the team played a bad pass and the opponent scored].” Knowing how good the opponent was may have indirectly intimidated the team enough to lower the team’s confidence of its own strengths. Sport Psychologist 4 (handball) similarly explained that the players overestimated the opponent and underestimated their own capabilities: “Respect for the opponent was too high. We were about to play against one of the best teams in the world and expectations about having to do something special were too high. It seemed like they went into the game with that idea in their head of ‘We can only win if somehow everything works out well for us.’” In this case, respect for the opponent was so high that the team did not believe they could win without some external factors (i.e., luck or poorer performance by the opponent) in their favor. This is consistent with Rotter’s (1966) external locus of control – an individual’s assumption that life is based on external factors, which cannot be influenced. The respect for the opponent and their subsequent external locus of control could have made them more vulnerable for a team collapse when the critical event occurred. This subsequent external locus of control could possibly also be related to self-handicapping (Jones and Berglas, 1978), as the team seemed to not believe in a win and may have provided excuses for the outcome of the game prior to its start.

In addition to the antecedents reported by Wergin et al. (2018), a *lack of self-confidence* was mentioned. Coach 7 (soccer) explained that his team was lacking self-confidence due to prior poor performance: “It simply didn’t work that well during the last weeks regarding the results and that’s why self-confidence was missing. The self-confidence and the mental toughness and the trust in the strength of the team was missing and then such a process [a team collapse] can easily happen.” Sport Psychologist 3 (handball) described how the apparent lack of self-confidence of his team made it more vulnerable to a critical situation occurring during the game: “The trigger clearly was this key situation but there is more to this than meets the eye. Why can such a situation be so effective? I believe because the team did not play with the same self-confidence in this game as they did in prior games and that they never gained an inner security, this feeling of ‘We know what we’re doing, this works.’ There was always this latent insecurity and I believe the team did not feel secure prior to the game already.” Insecurity was also reported to play a role in team collapse situations in existing literature (Wergin et al., 2018), but it was revealed to be an outcome of a critical team collapse event. In this study, a lack of self-confidence was reported to be present prior to the critical team collapse event. A similar association between self-confidence and performance under pressure was found in several studies on individual choking (e.g., Baumeister et al., 1985; Craft et al., 2003; Woodman and Hardy, 2003; Otten, 2009; Hill and Shaw, 2013), positive psychological momentum (Jones and Harwood, 2008), and team resilience (Morgan et al., 2013, 2015; Decroos et al., 2017). It is, however, a new finding in the research on collective team collapse. Furthermore, the presented quotes indicate that other antecedents, such as respect for the opponent, may have caused a lack of self-confidence, making the teams more vulnerable to a critical event on the court. This association will be discussed further in the section “General Discussion.”

### Critical Events

The most common critical event, reported by coaches and sport psychologists, was an *unforced error accumulation* within the team. Coach 3 (volleyball) described how cumulative failures contributed to perceptions of helplessness in his team: “We couldn’t score a single point. Not true, we scored one point, I believe we served when it was 14-9 [for us] and the serve was out or [hit the] net. Nothing worked anymore, really nothing! Then it was 14-10 and we gave away point after point, although we had good and prestigious players on the court. We didn’t know what to do.” It appears that the team was unaware of what was happening and what to do to recover from the collapse. Coach 7 (soccer) explained in more detail how, in his view, errors within his team changed the team’s psychological state: “It happened relatively sudden and it was an important mental situation for the team. We scored and we were in the lead and clearly superior to the other team. Then, our goalkeeper made a mistake and the whole team started to produce failures. I think this is what often leads to a team collapse. If it does not work well for the team, trust and the mental strength get lost. They start to doubt that they can get back into the game.” It appears that the accumulation of the mistake of the goalkeeper and the mistakes of the team that happened right after have caused a feeling of insecurity in players, which confirms the findings of Apitzsch (2009a) and Wergin et al. (2018). Furthermore, the errors seemed to have increased despair in the team, which will be elaborated further in the “Cognitive Outcomes” section.

Coaches and sport psychologists reported *key player collapse* to be a critical event. Coach 5 (basketball) clarified how a key player experiencing choking affected the rest of the team: “If one, an important player or a key player, who usually always carries the others along, if he collapses or has a bad day, which is totally normal, he pulls the team down… Like I said this is a team sport and if someone doesn’t pull through, he pulls down a second one and then then this negative spiral, a collapse, can happen very fast.” This statement illustrates how other players orient themselves toward the key players and experience a decrease in self-efficacy if a key player chokes (Apitzsch, 2009a; Wergin et al., 2018). Sport Psychologist 4 (handball) reported another possible association between a key player choking and collective team collapse: “The key situation was that they [the team] missed three seven-meters [shots] in a row but it was even more important that the key player was the one who missed the first seven meter [shot] and what happened then was that responsibility was continuously shifted to the next player.” In this example, it appears that the team was so dependent on the key player scoring that they were unsure about what to do or how to compensate for the key player’s underperformance and kept shifting responsibility to other teammates. This connection between key player collapse and the transfer of responsibility to other players adds to existing team collapse literature.

Coaches and sport psychologists further confirmed *scoring of the opponent* to be a critical event. Sport Psychologist 2 (volleyball) explained how his team started to collapse after both teams were fighting for an important point during a long rally and the opponent scored: “It was a combination of an unexpected point gap and several losses of points. In this situation, a point was lost, they fought for very hard, and then you got the impression that the willpower of the team was exhausted and the concentration was gone and then the set was lost although it could have been won.” In his description, it seems that just the single point the team worked hard to win but the opponent scored led to extreme disappointment for the players, which may have caused a change in the team’s mind-set and triggered the collapse. Sport Psychologist 3 (handball) similarly described that scoring of the opponent changed the team’s cognition: “Instead of leading by three goals, it happens fast, counterattack, in the lead with only one goal, and you realize how the team changes into a different mode and in the end loses the game… They could not gain back their solution-oriented thinking, which could have allowed them to win.” It appears that scoring of the opponent changed the teams’ view of the situation and interfered with their performance, allowing a team collapse to happen. The team seems to have lost its solution-focused thinking after the opponent scored and turned into a problem-focused mode of thinking, worrying about their opponent’s good performance instead of focusing on their own performance goals. Scoring of the opponent was found to trigger a team’s underperformance in existing research (e.g., Jones and Harwood, 2008; Apitzsch, 2009a; Wergin et al., 2018). These studies, however, did not explain the connection between loss of points and a change in the team’s mind-set.

Furthermore, coaches reported that a perceived wrong *referee decision* may have started their team’s collapse. For example, Coach 1 (field hockey) explained: “There were two controversial referee decisions made against us that led to two goals of the opponent. That means the referees were in the focus of the team collapse and our captain went to one referee and if the captain starts yelling at the referee already, it doesn’t take long until the others do the same.” Apparently, the perceived wrong referee decision caused anger of the team captain and made him show his aggression against the referee. The anger and frustration may have transferred to other players, who also became angry and did not focus on the game anymore. This corroborates other researchers’ findings (e.g., Jones and Harwood, 2008; Wergin et al., 2018).

In addition to the critical events reported by Wergin et al. (2018), coaches described *game interruption* as a critical event causing a team to collapse. The *game interruption* due to an injury (of own teammate or opposing player) changed the team’s performance as Coach 4 (volleyball) explained: “One of the opponent’s players got injured, there was an interruption of the game, he was also whining a little and after that nothing worked anymore within our team. It was the typical case, we could have played so much better but… after this happened nothing worked anymore on our side.” Even though it was not a teammate who was injured, the team somehow lost focus due to the interruption and was unable to keep up the same level of performance once the game recommenced. Apitzsch (2009a) also reported that a time out called by the coach as well as the coach’s behavior during this time out interrupted the play of the team and provoked a team collapse. This critical event constitutes a new category in the process model of collective sport team collapse that will be discussed later on.

### Outcomes Maintaining Team Collapse

Outcomes maintaining team collapse specifically refer to factors that occurred after a critical event and contributed to the development and continuation of the team collapse. Like athletes, coaches and sport psychologists found that a critical event happening on the court had an impact on the team, which may have led to various cognitive, affective, and behavioral processes within the team. These cognitive, affective, and behavioral changes may have led to further mistakes and underperformance that maintained the team collapse. It appeared that cognitive and affective outcomes preceded behavioral outcomes in the coaches’ and psychologists’ descriptions; hence, they are presented first.

#### Cognitive Outcomes

Cognitive outcomes include thoughts, thought processes, and perceptions, which hindered the team from gaining back their previous performance level. One of these factors was pressure that was induced by the underperformance of the team and the need to recover from the team collapse in order to stay in the tournament. Sport Psychologist 4 (handball) explained how *pressure* to score was induced: “We had 10 more minutes to go and the team was behind by five points and many already saw their dream vanishing. And they [the players] calculated something like: ‘Now there are only 10 minutes left, if we don’t score five times, we won’t participate in the championship.’ And this is exactly what shouldn’t happen.” Unlike perceived pressure due to the importance of a game (in the “Antecedents” section of this paper), the sport psychologist described how pressure on the team increased due to the team’s underperformance and being behind on the scoreboard. This perceived pressure to perform better may have hindered the team even more in gaining back their previous performance level and thus maintained the collapse. The athletes in Wergin et al.’s (2018) study also reported increased pressure induced by the perceived urgency to overcome the team collapse.

The team collapse itself and the pressure induced by it seemed to raise *insecurity* in players. Sport Psychologist 3 (handball) explained how the team was unable to gain an inner security due to the persistent team collapse: “The team went into an uncontrolled mode. There was a latent insecurity present. The first half wasn’t good already, we were behind and luckily finished the first half tied, which we didn’t deserve with this performance… And I think this underperformance was what caused the feeling of insecurity.” The latent insecurity appears to be a result of the team’s bad performance and may have led to unrest and a loss of perceived team control, which prohibited the team from returning to their regular performance level, potentially exacerbating the team collapse. While this connection between insecurity and performance (Morgan et al., 2013, 2015; Decroos et al., 2017) and between insecurity and team collapse (Jones and Harwood, 2008; Apitzsch, 2009a; Wergin et al., 2018) has been presented before, Coach 5 (basketball) further mentioned a connection between his behavior and the perceived insecurity of the team. He explained how a coach could also increase insecurity in players through his/her behavior during a team collapse: “If you become nervous outside and start scratching your head, you create an insecurity in the players. If you as a leader are insecure, it becomes difficult. As a coach you have to send out positive and confident signals to the team.” As a perceived leader, the coach’s behavior can affect the team. Although several studies have emphasized the impact of coaching behaviors on sport teams (e.g., Loughead and Hardy, 2005; Myers et al., 2005), the influence of a coach’s behavior on a team’s performance, or feeling of security, has not been reported in existing literature.

Coaches and sport psychologists further reported that their team suffered from a *lack of accountability*. The players appeared not to take responsibility for scoring anymore after the critical team collapse event happened. Coach 3 (volleyball) stated: “There was no one there saying ‘Give me the ball, I’ll do that’ or simply ‘Give it to me, I’ll block it down. No one took initiative or gave the others an idea of what to do.’” Sport Psychologist 4 (handball) found that especially key players did not take enough responsibility: “When the key player missed [the seven meter shot], responsibility was passed on to the next and the next [player] and it became absurd who threw seven meters… Some key players did not assume the responsibility that you would wish they did. There were two more seven meters after that and I would have expected them [the key players] to throw them.” Wergin et al. (2018) explained the shift of responsibility between players might be associated with the social loafing process that can occur in groups (Karau and Williams, 1993). Another possible explanation for the above quote might be the diffusion of responsibility in groups where the presence of others lowers the feeling of responsibility in individuals when facing a difficult situation (Darley and Latane, 1968). Morgan et al. (2015, 2017) in this context report that collective accountability contributes to team resilience, especially when the team faces setbacks. They further found that certain leadership strategies could enforce accountability and increase performance. Athletes within Wergin et al.’s study also mentioned that the reason for the transfer of responsibility could be anxiety about being responsible for the next mistake. An interesting finding of the current study is that coaches stated that this especially applied to key players, which may be problematic for the team, as players tend to orient greatly toward key players (Apitzsch, 2009a; Wergin et al., 2018).

Similar to athletes in the Wergin et al. (2018) study, coaches and sport psychologists found that increasing *despair* and perceived helplessness within the team were an outcome of team collapse that fostered its maintenance. Sport Psychologist 1 (soccer) stated that players of the team reduced their effort because of the team’s underperformance and their disbelief in their ability to change the situation: “Some players did not really participate in the game anymore. In soccer as it is played today, the play completely depends on every player… The game only took place on a part of the field, everyone was very situation-oriented and then a few stopped playing as if to say ‘the others collapsed already and I don’t believe in the victory anymore.’” Sport Psychologist 4 (handball) described that his team did not believe that they would be able to change the game outcome anymore: “It seemed like they thought a foreign force had conspired against them and that they thought ‘Nothing will work anymore anyway.’ And in accordance with this the players began to hang their heads.” This belief of not being able to change anything is a very typical outcome of a team’s underperformance (Jones and Harwood, 2008; Hill and Shaw, 2013; Wergin et al., 2018) and is likely related to learned helplessness (Seligman, 1975), because the team seems to believe to have lost the ability to change their situation.

A new cognitive outcome reported by coaches and sport psychologists in this study was the *failed expectations* of the team. Sport Psychologist 2 (volleyball) explained: “The team that I supervised should have been clearly in the lead, but they weren’t and I believe this was a moment that caused confusion in the team… They were so confused and the coordination between players, which is crucial in volleyball, was gone, and you felt that everyone was busy dealing with himself. I believe the focus on the general tactics got lost because everyone was busy dealing with the unexpected situation.” It seems that the team’s high expectations prior to, or during, the game triggered a disappointment that their expectations were not being realized, because the team was not prepared for this unexpected and insurmountable lead by the opponent. Although the disappointment of prior expectations is known to be an outcome of individual choking under pressure (Hill et al., 2011) and the underperformance of a team (Jones and Harwood, 2008), it has not been reported as a factor maintaining collective team collapse. The maintenance of the collapse, due to disappointed expectations, can possibly be explained through Personality Systems Interaction (PSI) Theory (Kuhl, 2000). According to PSI Theory, negative affect, resulting from frustration, interferes with access to one’s capacities. Building on this, the disappointment of expectations of a team after a critical event might increase negative affect, which hinders the players in retrieving their full performance capacities, and, therefore, maintain the underperformance.

Sport psychologists also described an *actionist atmosphere* to be spreading within the team a result of the team collapse situation. This actionist atmosphere was defined as players’ perception of an urgent need to score in order to end the collapse, which seemed to lead to rather ineffective moves. Sport Psychologist 4 (handball) stated: “There was this actionist atmosphere developing. You had the feeling they wanted to score by will and force… they thought ‘Now that it [the game] doesn’t go well, we have to do even more,’ and I believe that was too much.” According to Sport Psychologist 4, this perception of a need to do more to change the situation did not improve performance but instead maintained the collective team collapse. The absolute determination of the team to score seemed to inhibit their performance even more. This finding is new to team collapse literature and has not been reported in research related to the underperformance of individuals or teams so far.

Sport psychologists also described that in their perception, an *individualization* of players maintained the team collapse. Individualization was described as a loss of coordination between the players. It seemed that every player acted on his or her own instead of working together after a critical event. Sport Psychologist 2 (volleyball) explained: “Regarding the process, I would say there was a shift of focus from the whole team, from the interaction [of players] to the individual. Everyone was on his own, occupied with his emotion, with his frustration about the situation and maybe also with other thoughts related to this. The coordination between players got lost and everyone was on his own with this frustration.” The critical event seemed to have changed the team’s perception of itself from working in unison to becoming more individually focused, which appeared to be accompanied by a decrease in coordination between players that fostered the maintenance of the collapse. Apitzsch (2009a) similarly reported that team processes, such as interaction and cooperation, tended to break down during a team collapse and that team players started to play as individuals rather than as a team. He further explained that the performance of the players declined even more through this individualization and the stress that went along with it, resulting in chaos and defeat. Morgan et al. (2015, 2017) further explain that coordination between players is a crucial factor for team resilience and thus especially important when a team faces a stressful situation.

Another new outcome mentioned primarily in sport psychologists’ perceptions was the development of a *prevention orientation* or a shift in focus from winning to “not losing.” Sport Psychologist 1 (soccer) said: “It should have been clear that we have a goal and not that ‘It’s our job to defend something.’ And suddenly it was all about not letting the catastrophe happen and the goal orientation transformed into a prevention orientation. And in combination with small mistakes of usually very stable players, the others [team mates] suddenly lost orientation and weren’t able to work that well in defense anymore.” This switch from an aspirational goal to a focus on prevention likely changed the behavior of players on the court to a defensive or less offensive play, making it even harder for the team to score and overcome the collapse. This change of a team’s mindset has not been reported in team collapse literature. Apitzsch (2009a) reported that, due to the collapse, the offensive play of the team decreased significantly, which may be a result of a developing prevention orientation.

#### Affective Outcomes

Affective outcomes are changes in emotions and feelings in the team that prohibit it from returning to the original performance level. The affective outcome of *negative emotional contagion* was mentioned by coaches and sport psychologists. Coach 1 (field hockey) explained that the key player’s mood transferred to other players: “In my opinion, a team collapse happens if key players are, I don’t know how to say it, for example if they are dissatisfied with the game and then the [other] players adapt to the key player and the negative mood transfers to the whole team.” Sport Psychologist 3 (handball) emphasized how the team was unable to stop the spread of anxiety among the team: “There was not much time left to play and to return to our initial performance level… The self-confidence vanishes and anxiety spreads within the team, and the exact opposite happens in the opponent team. Within the last eight minutes of play, this atmosphere is dominant in the team and they can’t get out of there.” Existing research reported a connection between individual emotion and emotion of the team (George, 1990; Totterdell, 2000) as well as a connection between the spreading of negative emotions and a performance decrease of the team (Kelly and Barsade, 2001; Apitzsch, 2009a; Boss and Kleinert, 2015; Wergin et al., 2018). This transfer of emotion among team members has also been referred to as negative emotional contagion (e.g., Totterdell, 2000). Apitzsch (2009a) further explained that the negative emotional contagion has a negative impact on athletes’ cognition and leads to negative thoughts, which seem to foster a maintenance of the team collapse (Wergin et al., 2018). Complementary to this, Morgan et al. (2017) found that positive emotions are related to team resilience as well as to a team’s performance.

As Sport Psychologist 3 (handball) pointed out, *anxiety* seemed to play a crucial role in maintaining collective team collapse. Coaches and sport psychologists recognized the outcome or consequences of anxiety, whereas Psychologist 3 (handball) described the origin of the anxiety that spread in his team: “From that positive atmosphere, that certainty, from this offense situation ‘We can achieve something’ it [the atmosphere] shifts to anxiety: ‘Oh shit, now we’re in the lead with only one goal’ and then the others get into the flow, it’s a classical shift of momentum.” It seems that scoring of the opponent caused a fear of losing the game within the team, which further appears to be associated with or a consequence of the earlier mentioned cognitive shift from goal to prevention orientation. Coach 5 (basketball) similarly described: “You realize it immediately, that players drop shoulders and become anxious ‘Hopefully I don’t make the mistake’ and ‘hopefully it’s not my fault’ and things like that, you can see that from the outside.” Apparently, players were afraid to make further mistakes and feared to be held responsible for the team collapse. Researchers have found that a fear of failure is associated with increased anxiety and decreased performance (i.e., choking under pressure) in individuals (Jones and Harwood, 2008; Hill et al., 2009; Otten, 2009; Gucciardi et al., 2010) as well as performance decrements in teams (Apitzsch, 2009a; Wergin et al., 2018). It is assumed that pressure, the desire to perform well, and a lack of confidence in one’s abilities might lead to anxiety associated with failure, which prohibits a return to a regular performance level and maintains the team collapse (Wergin et al., 2018).

Coaches and sport psychologists further reported that *anger and frustration* were negative emotions associated with the team collapse in their opinion. Coach 7 (soccer) explained that failure of players and the externalization of reasons for this failure may have caused frustration and anger within his team, which appear to increase the negative performance and maintain the team collapse: “From the perspective of a coach I can say that it [the team collapse] is a process that maintains and fosters itself. When the game becomes worse, you get the feeling that the players always try to find excuses for their failure and then frustration about failures of team mates or about own mistakes increases.” Sport Psychologist 2 (volleyball) explained how frustration then led to individualization within the team: “Everyone was busy dealing with their emotion, their frustration about the situation, maybe also with other thoughts related to this and then the coordination between the players got lost and everyone was dealing with the frustration on her own. The same thing happened with the coach.” The frustration that appears to increase in players and coach also seemed to interfere with the coordination between the players and caused them to withdraw from each other. The results are consistent with the findings of Apitzsch (2009a) and Wergin et al. (2018) but provide some additional information about coaches’ and sport psychologists’ perceptions on the role that frustration and anger play within the cascade of causes of collective team collapse.

#### Behavioral Outcomes

Behavioral outcomes are changes in actions resulting from cognitive and affective changes that also foster a maintenance of the team collapse. One behavioral factor reported by coaches and sport psychologists was *decreased performance contagion*, which was defined as poor performance transferring among players. Sport Psychologist 1 (soccer) explained: “It [the poor performance] was especially related to midfield and defense and nothing fit together anymore and that means, for a team that moves as a collective team, everyone suddenly slumped.” The decrease of performance of several players seemed to negatively influence the team’s collective interplay and therefore led to a performance collapse of all players. Researchers (e.g., Boss and Kleinert, 2015; Wergin et al., 2018) also reported decreased performance contagion, where poor performance of a key player mainly transferred to other players, who oriented themselves toward the key player and tended to adapt to the mood or performance of that key player. Through the quote of Sport Psychologist 1, a perceived underlying link between the performance decrease and the interplay of the team is presented, where the team underperforms collectively due to a disturbance in the play system evoked by individual players.

The behavioral outcome of *cautious play* was mentioned by sport psychologists and coaches. Coach 3 (volleyball) for example explained: “I believe we became too cautious” and Coach 4 (volleyball) stated: “My personal opinion is that when the team collapse happened, we weren’t playing sovereign [superior to the other team] anymore, we were insecure due to two bad actions before.” Coach 4 described how poor play of her own team caused players to feel insecure, which seemed to provoke a cautious or defensive playing behavior. Cautious play has been found to be an influencing factor in previous studies (Wallace et al., 2005; Wergin et al., 2018), which underscores the notion that one’s underperformance (critical event) may lead to a change in other players’ cognition (feeling insecure), which might affect playing behavior (playing cautiously).

Contrary to this, sport psychologists also mentioned that some teams tended to play more frantically, being in a *hectic rush*, after a critical team collapse event. Sport Psychologist 4 (handball) in this context explained: “You have to imagine that the goal keeper passes and then they wanted to perform an attack, then a bad pass happens and after receiving a goal, they played in a hectic rush and tried to make a fast counter attack that found a hasty end.” This sport psychologist suggested that receiving a goal seemed to induce pressure or gave the players the feeling that they had to score fast in order to end the team collapse, which led to a maintenance of the bad performance. Jordet and Hartman (2008) similarly reported that soccer players in penalty shootouts took shorter times to prepare their penalty shots as an avoidance coping technique to deal with the high-pressure situation and showed individual choking behavior as a result. A hectic rush was also found to be important in maintaining team collapse by Apitzsch (2009a) and Wergin et al. (2018). We suspect that the feeling of an urgency to score, which is also described by Sport Psychologist 4, may represent the cognitive experience a team goes through before showing a hectic behavior on the field. This will to score in order to end the collapse is assumed to be what we described as an actionist atmosphere in the cognitive outcomes. It appears that increased pressure (e.g., due to scoring of the opponent) may create an actionist state of mind, which might manifest through a hectic playing behavior.

*Limited communication* was reported to play a crucial role in maintaining team collapse by coaches as well as sport psychologists. Sport Psychologist 2 (volleyball) explained: “I believe that the contact between rallies was less emotional, less contact. The players in this situation as well as in other situations where it didn’t work, had less contact with each other. They became more separated and did not celebrate anymore in between rallies when they scored a point.” Interestingly, Sport Psychologist 2 made a direct connection between the lack of communication and the individualization of the players, whereby it seems that a lack of communication is the behavioral and thus observable part of the individualization happening in the team. Sport Psychologist 1 (soccer) further mentioned that key players in particular should talk to their teammates and direct an appropriate course of action, but did not communicate much anymore during the collapse: “When a difficult situation occurs, the players themselves have to communicate a strategy. The coach cannot communicate that from the side of the field. Key players have to take half a minute after a goal to talk to the others ‘Hey, let’s do this and that’ to shortly dispute on the field. But they were so confused that no one talked to the other.” This statement illustrates that the lack of communication seems to involve key players as well and that they may play a significant role regarding the decrease of communication within the team. This is not surprising, since research highlights the importance of communication for a team’s performance (Apitzsch, 2009a; Morgan et al., 2013, 2017; McEwan and Beauchamp, 2014) and shows that key players tend to promote team efficacy (e.g., Bandura, 1977; Spink, 1990; Carron and Hausenblas, 1998). However, the connection between key players, communication within a team, and team collapse has not been reported in existing literature.

The maintaining factor of *blaming others* was perceived by coaches. Interestingly, they did not only state that players started to blame each other during a team collapse but further explicitly stated that this was enhancing the effect of the team collapse. Coach 7 (soccer) in this context said: “You should never blame someone else, a team mate for failure, especially not during a team collapse. In my opinion this is crucial. If you are busy blaming someone else for it, even if it’s the referee or the own player who did the mistake, or even someone external, this enhances the effect of the team collapse.” This finding supports the assumption by Wergin et al. (2018) that the behavioral outcome of blaming others for failures contributes to the maintenance of team collapse. Morgan et al. (2013, 2015) similarly explain that a culture of no blame can foster team resilience and is important when failure happens within a team.

Besides the behavioral categories of the original process model of collective team collapse (Wergin et al., 2018), several new factors emerged from the coaches’ and sport psychologists’ perceptions within this study. One of these was an increasing *immobility* of the players on the court. Coach 4 (volleyball), for example, described how the players would not move anymore after the other team had scored: “It was out of the sudden that, nothing worked anymore, no reception, no movement towards the ball, no extra movement, no reaction, they’re just looking at each other, not knowing what to do.” The shock and disbelief about the deficit in points and maybe also the fear of losing the game seemed to keep players from moving toward the ball. Immobility constitutes a new finding in team collapse research.

Another behavioral outcome, which was novel in sport psychologists’ interviews, was *play system collapse*, described as the collapse of the team’s game structure. Sport Psychologist 4 (volleyball) explained: “From an outside perspective I would say that there was this moment when the opponent scored and after the opponent scored, they couldn’t get a structure back into the game and that made them lose the game and fail utterly.” Sport Psychologist 2 (volleyball) explained in more detail how the loss of points caused confusion in his team leading to a lack of coordination, increased individualization, and ultimately a loss of the play system: “There were some unfavorable losses of points and a hard-fought rally, which got lost, and then they got confused and the coordination between players, which is crucial in volleyball, got lost and everyone was dealing with himself. I believe the general tactics got lost because everyone was busy dealing with that unfamiliar or forbidden situation.” This breakdown in the playing structure of a team due to a team collapse is a novel finding in the understanding of team collapse processes.

**Figure 2:**
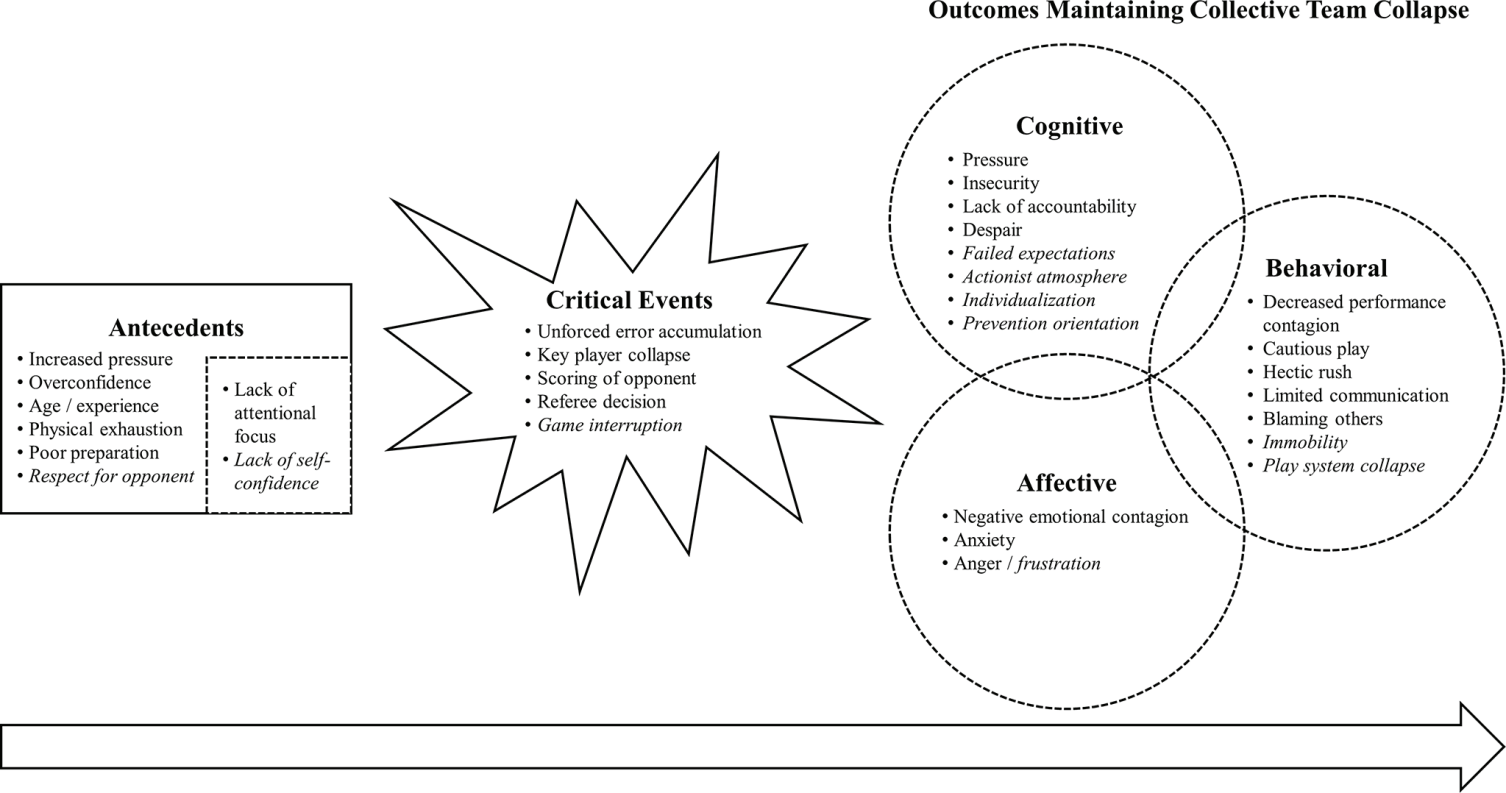
Adapted process model of causes of collective sport team collapse. Alterations between Figure 1 and this figure have been marked. New categories are illustrated in italic type; changes in the structure of antecedents and outcomes are marked through dotted lines.

Several new categories were perceived by coaches and sport psychologists in the current study in addition to the categories of Wergin et al.’s (2018) study. Figure 2 illustrates an integrated process model based on Wergin et al.’s (2018) theoretical framework and the data of this study. Compared to the initial process model of collective sport team collapse (Figure 1), the adapted process model (Figure 2) distinguishes between prior and posterior antecedents, whereby the posterior antecedents emerge from the prior antecedents. While critical events remain the same between initial and adapted process model, the order of outcomes maintaining team collapse changed in the adapted model. Because coaches and sport psychologists described that behavioral outcomes would emerge from cognitive and affective outcomes, behavioral outcomes are displayed last and after cognitive and affective outcomes. The data informed model is supposed to give a broad overview of the temporal process of causes of collective team collapse by incorporating athletes’ (Wergin et al., 2018) as well coaches’ and sport psychologists’ perceptions. Categories within the model should function as examples to illustrate the interplay of causes of team collapse and do not provide a complete representation of every possible combination of factors. It should further be mentioned that, similar to the process model of Wergin et al. (2018), the adapted process model of collective sport team collapse (Figure 2) illustrates the results of the analysis and interpretations of the data of the current study, rather than general characteristics of the team collapse phenomenon. Results are presented in a linear fashion to account for participants’ descriptions of a temporal difference in the components of the model (i.e., antecedents, critical events, outcomes). This linear presentation is not supposed to contradict a cyclical and dynamic process that is in some ways typical in team dynamics research. A cyclical process may still occur within our linear framework, but was not further investigated during coaches’ and sport psychologists’ interviews in this study.

## General Discussion

The goals of the current study were to explore coaches’ and sport psychologists’ perceptions of causes of collective team collapse, to qualitatively compare their perspectives to athletes’ perceptions, and to explore whether coaches’ and sport psychologists’ perceptions would support the process model of causes of collective sport team collapse (Wergin et al., 2018).

The general structure of Wergin et al.’s process model was also found in data collected in this study. Perceived antecedents and critical events leading to team collapse were similar between the three participant groups (i.e., athletes, coaches, and sport psychologists) in the current and Wergin et al.’s (2018) study. Nevertheless, a *lack of self-confidence* was brought up as a new antecedent and *game interruption* as a new critical event by coaches and sport psychologists in this study, compared to Wergin et al. It appears that antecedents themselves are temporal and do not happen concurrently, which is why they are presented in a temporal order in Figure 2. A *lack of attentional focus* and the *lack of self-confidence* seem to be internal antecedents of the players that result from rather external antecedents such as *respect for the opponent* and thus temporally follow as secondary antecedents.

While perceptions of coaches and sport psychologists in the current study supported the framework of affective, cognitive, and behavioral outcomes maintaining team collapse, they also mentioned several new categories, in addition to the ones reported by athletes in the initial process model (Wergin et al., 2018). Categories that emerged from the quotes included *failed expectations*, an *actionist atmosphere*, *individualization*, and a *prevention orientation* of the team as new cognitive outcomes, and *immobility* and *play system collapse* as new behavioral outcomes. Regarding the temporal order of affective, cognitive, and behavioral outcomes within the process model of causes of collective sport team collapse, participants in this study explained that behavioral outcomes were a result of affective and cognitive outcomes, although they could not specify whether affective or cognitive outcomes preceded. Thus, behavioral outcomes represent the final temporal outcome in the sequence of causes of team collapse (Figure 2). Nevertheless, coaching staff are not as involved in the team collapse process as the athletes on the field and therefore may automatically focus more on the behavioral component of outcomes of team collapse, since it is the most visible and accessible.

In the context of outcomes maintaining team collapse, coaches also tended to especially see behavioral factors, for example *immobility* or the *blaming of others*, as essential for maintaining team collapse. In contrast, sport psychologists especially reported cognitive factors, such as *individualization* or a *lack of accountability* between the players, to maintain team collapse. Accordingly, coaches found behavioral outcomes to be more relevant during a team collapse situation, while sport psychologists focused more on cognitive outcomes. This finding may be explained by the different focal points the two participant groups gain during their education and career. While psychologists’ area of responsibility typically deals with cognitive processes, coaches tend to focus on the more salient area of action and behavior, which they likely perceive they can influence. While describing these processes, sport psychologists appeared more neutral and showed less emotional attachment to team collapse events than athletes and coaches. They also used social psychological theories to explain how underlying processes of team collapse evolved; for example, one sport psychologist explained that, due to the scoring of the opponent, the goal orientation of his team to win the game transformed into a prevention orientation to prevent the loss of the game. Compared to coaches, sport psychologists are not necessarily former athletes of the sport. In contrast, most coaches are former players (e.g., Rynne, 2014; Mallett et al., 2016) and their own experience with collective team collapse might shape their perceptions of the phenomenon. They may include their own experiences from their time as an athlete in their report of collective team collapse.

It further needs to be noted that several categories of the initial process model (e.g., *lack of attentional focus, age and experience, poor preparation, insecurity, lack of accountability, limited communication, blaming others*) as well newly described categories in this study (e.g., *lack of self-confidence*, *individualization*) can also be associated with team resilience. Studies of Morgan et al. (2013, 2015, 2017) and Decroos et al. (2017) report contrasting factors such as *resetting focus, collective efficacy, thorough preparation, positive emotions, group accountability, frequent communication*, or a *no blame culture* as factors that can strengthen a team’s resilience when encountering stressful or challenging situations. By looking at team resilience and the factors described by coaches and sport psychologists in this study, it appears that team resilience may constitute a protective factor against collective team collapse. If a team manages to improve collective efficacy, game preparation, the group accountability, and communication in the team and to develop strategies to reset focus or establish a culture of no blame, they may be less vulnerable for stressful situations or critical events on the court and less likely to experience a collective team collapse. The interplay of team resilience and team collapse constitutes an interesting starting point for future research.

Overall, the present study was the first one to examine coaches’ and sport psychologists’ perceptions on causes of collective sport team collapse. It gives insights into perceptions of causes of team collapse in a variety of sports and adds new factors to the cascade of causes evoking collective team collapse. Based on these findings, it provides practical implications and offers new questions for future research. Compared to earlier studies in the field (Apitzsch, 2006, 2009a,b; Moesch and Apitzsch, 2012), it includes both male and female participants from different sport disciplines and contains information on the relations and interplay of the different factors involved in evoking a team collapse. It complements the findings of Wergin et al.’s (2018) study by two more perspectives (i.e., coaches and sport psychologists) and allows a 360° view on the phenomenon of collective team collapse.

### Limitations

A limitation to the current study is that the short colloquial description of team collapse that participants received prior to the interview might have influenced their descriptions of the phenomenon when they recalled their team collapse experiences. It is also noteworthy that the coaches in this study might have reflected on their previous playing experiences in discussing their perceptions of the phenomenon. Similarly, the sport psychologists may have included their theoretical knowledge about group processes and group phenomena in their descriptions of collective sport team collapse. Moreover, the study mainly included professional coaches and sport psychologists (and one semiprofessional coach) working in elite sports. To further address team collapse occurring in amateur sports, future research should include athletes, coaches, and sport psychologists of amateur leagues as well. Another point to be raised is that coaches and sport psychologists in this study were coaching in between national and fourth division. This difference could also indicate a skill difference.

### Future Research

Although this study provides new insights into coaches’ and sport psychologists’ perceptions of collective team collapse and its underlying processes, further empirical examination of this phenomenon is necessary to more comprehensively understand it. The present study only included professional coaches and sport psychologists working in a few elite sports. In order to further address team collapse occurring in amateur (and other) sports, future research should consider other settings and sports. It is suggested that both qualitative and quantitative studies are necessary to examine this phenomenon further. Specifically, a more nuanced understanding of the interdependency of the temporal factors (e.g., how they relate to each other) is necessary; that is, future studies might investigate the relations between affective, cognitive, and behavioral outcomes in more detail by including athletes, coaches, and sport psychologists in a focus group discussion about the associations between affective, cognitive, and behavioral outcomes maintaining team collapse. Sport-specific causes of collective team collapse should also be investigated, as it appears that especially bench coaching and non-bench coaching sports deal with different problems during a team collapse process. Moreover, after further empirical examination, possible interventions to minimize the risk of and the capability to disrupt a team collapse need to be developed and tested. Another interesting matter for future research to elaborate on might be to explore the interplay of team resilience and collective team collapse and to investigate whether or to what extent resilience can protect a team from experiencing a collective collapse.

### Practical Implications

Based on the findings of this study, we propose several practical recommendations and implications. Emotional regulation strategies and coaching of key players to intervene in team collapse situations have been reported as possible intervention strategies (Wergin et al., 2018) and would also fit within the results of the current study (e.g., to prevent *anger and frustration*). The newly reported cognitive outcome of *individualization* may be thwarted through key player actions in a team collapse situation. Furthermore, the new categories of *game interruption*, *play system collapse*, and *immobility* indicate that a team’s interplay is disturbed by a critical team collapse event. In these situations, another interruption during the team collapse might be helpful, which could involve calling a time-out or substituting a player to potentially reset the team, especially in bench coaching sports, in which these strategies are allowable. In sports where coaching during game play is difficult or impossible, half-time breaks could be used to intervene. It seems that, in order to be effective, interventions conducted during time-outs or half-time breaks should attempt to change the teams’ focus from a prevention orientation of “not letting the catastrophe happen” to the perception of opportunities and possibilities in the further course of play (goal orientation). Wergin et al. also suggest that establishing a culture of no blame and including resilience and pressure simulation trainings into practice might be helpful. These practical implications and recommendations, however, need to be examined in future research.

## Conclusion

Overall, athletes, coaches, and sport psychologists supported the view that team collapse was a phenomenon in their sport. Participants’ perceptions in the current study resembled the general structure of the process model of causes of collective sport team collapse and added some categories to the existing theoretical framework. In addition, two adaptations to the process model were discussed. Taken together, the results of this study add richness to the process model of causes of collective sport team collapse. The proposed model should encourage future research in this area and provide a systematic overview of the complex phenomenon of team collapse to athletes and practitioners in the field.

## Ethics Statement

The study did not involve any invasive or potentially dangerous methods and therefore, in accordance with the German Research Foundation (DFG) and the guidelines of the Department of Sport and Health Science at the Technical University of Munich, did not require formal ethical approval.

## Author Contributions

The original research is part of the PhD thesis of VW, supervised by JB. VW, CJM, CM, and JB contributed to the conception and design of the study. ZZ and VW performed data collection and analysis. VW wrote the first draft of the manuscript. CJM, CM, and JB wrote the sections of the manuscript. All authors contributed to manuscript revision, read and approved the submitted version.

### Conflict of Interest Statement

The authors declare that the research was conducted in the absence of any commercial or financial relationships that could be construed as a potential conflict of interest.
