# Supplementary material for: When You Watch Your Team Fall Apart – Coaches’ and Sport Psychologists’ Perceptions on Causes of Collective Sport Team Collapse
Source: Front Psychol. 2019 Jun 12;10:1331. doi: 10.3389/fpsyg.2019.01331 (PMC6581729; doi:10.3389/fpsyg.2019.01331)
Supplement: Supplementary file 1 [file Data_Sheet_1.PDF]

Interview Guide adapted from Wergin et al. (2018)

Short colloquial description of team collapse read out to the participant: "A collective team collapse is the moment or process, when the performance of your team unexpectedly decreases more than normal. It is the situation, when your team experiences a significant performance collapse during a competition / game. It is the moment or process when 'nothing works anymore' within your team during a specific competition / game."

0. Describe a team collapse that happened during the last 12 months while you were coaching.

(*If a person cannot recall a team collapse within the last 12 months*: Describe a team collapse that happened during the last 5 years while you were coaching.)

Questions about the Team Collapse

1. What kind of game/competition was it?
2. At what point within the game did the team collapse occur?
3. How long did the collapse last?
4. How many players were involved?
5. What role within the team did the involved players fulfill?
6. What happened within the team during the team collapse?
7. Describe the atmosphere within the team during the course of play.

Impact of Team Collapse on Players and Game

8. What happened within the team during the team collapse?
9. Describe the atmosphere within the team during the course of play.
10. To what extent did the team collapse influence the further course of play?
11. To what extent did the team collapse influence the next training / game?
12. How do you react to team collapse instantly and after the game?

Influencing Factors of Team Collapse

13. In your opinion, what were the influencing factors for the team collapse?

14. Is there anything else you would like to mention regarding the topic of team collapse?
